# An Advanced-Stage Encapsulated Papillary Breast Carcinoma in a Male: A Case Report

**DOI:** 10.1155/crip/6518104

**Published:** 2025-08-28

**Authors:** Elyssa Glassheim, Jain Zhou, Stephanie Fine, Nadja K. Falk, Mary Torrez

**Affiliations:** ^1^Department of Pathology, University of New Mexico Health Sciences Center, Albuquerque, New Mexico, USA; ^2^Department of Surgery, Division of Surgical Oncology, University of New Mexico Health Sciences Center, Albuquerque, New Mexico, USA

**Keywords:** breast, encapsulated papillary carcinoma, male, metastatic breast carcinoma, papillary carcinoma

## Abstract

Encapsulated papillary carcinoma (EPC) is an invasive carcinoma which shows papillary architecture within a thickened fibrous capsule. Multiple studies have shown that this tumor follows an indolent course with excellent prognosis, and as such, it is recommended that it be staged as in situ lesions. It is an uncommonly encountered tumor most often diagnosed in postmenopausal females. As breast cancer in males is overall rare, available data on diagnosis, management, and outcomes of EPC in males is limited. Typically, cases of EPC that present with advanced stage and/or lymph node metastases show an associated invasive process. We present a case of pure EPC in a male patient with associated skin ulceration and positive lymph nodes, leading to a final stage of ypT4bN1a. The present report underscores the indolent nature of EPC, even when diagnosed at an advanced stage.

## 1. Introduction

Male breast cancer is rare, representing < 0.5% of male cancers and 1% of all breast cancers. Male breast papillary lesions are uncommon and consist of a heterogeneous group including benign, carcinoma in situ, and invasive carcinomas. Four percent of breast carcinomas in men are of the papillary subtype [1]. Encapsulated papillary carcinoma (EPC) is uncommon, particularly in males, and is generally believed to have a good prognosis.

EPC is a localized papillary carcinoma with fibrovascular fronds that typically grow in a cystic space and are surrounded by a fibrous capsule. EPC characteristically lacks a myoepithelial cell layer both within the fibrovascular cores and at the periphery of the tumor, a finding which, along with its generally indolent course, has in the past prompted discussion as to whether this disease represents an in situ or invasive process [2–6]. When there is an invasive component, it is currently classified as an indolent form of invasive carcinoma, and axillary lymph node and distant metastases are rare [3, 7].

If invasion beyond the fibrous capsule is absent, the 2022 World Health Organization (WHO) classification of breast tumors recommends classification as DCIS (ductal carcinoma in situ), also known as Tis, rather than invasive carcinoma and grading according to its nuclear grade; if invasion beyond the fibrous capsule is present, the area of the invasive cancer should be used for T stage. When an invasive component is present, it most frequently takes the form of invasive ductal carcinoma (IDC)/invasive breast carcinoma of no special type (IBC-NST) and can often have mucinous features [8].

For noninvasive EPC, complete surgical excision with negative margins is the recommended treatment. In EPC confined to the capsule without associated invasion beyond the capsule, the prognosis is very favorable with survival of > 95% at 10 years [9]. Advanced stage at presentation for EPC is highly unusual and is typically only observed in cases with an overt invasive component [10].

## 2. Case Presentation

**Figure 1:**
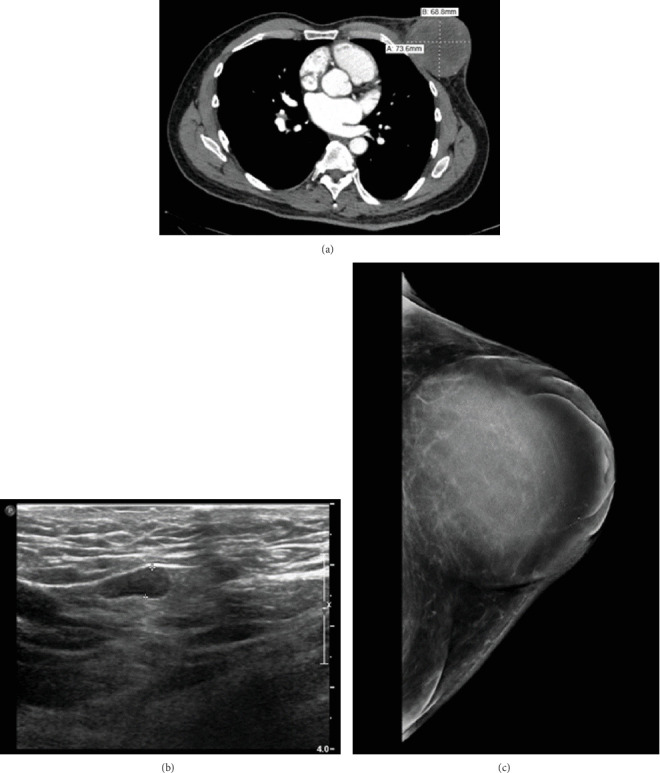
(a) CT shows a soft tissue mass along the left anterior chest wall measuring 7.4 × 6.9 cm. (b) US shows abnormal left axillary lymph nodes with cortical thicknesses measuring 5 mm. (c) Mammogram shows a 7.1 × 6.9-cm solid mass in the left breast centered at 12:00 position 4 cm from the nipple.

A 61-year-old previously healthy male presented emergently with pain and swelling of his left chest/breast. Although present for several years, the lesion recently demonstrated rapid growth and, ultimately, skin ulceration. Computed tomography (CT) revealed a 7.4-cm left chest wall mass (Figure 1a). An ultrasound (US) (Figure 1b) and mammogram (Figure 1c) confirmed suspicious left axillary lymph nodes and a 7.1-cm solid mass, respectively.

**Figure 2:**
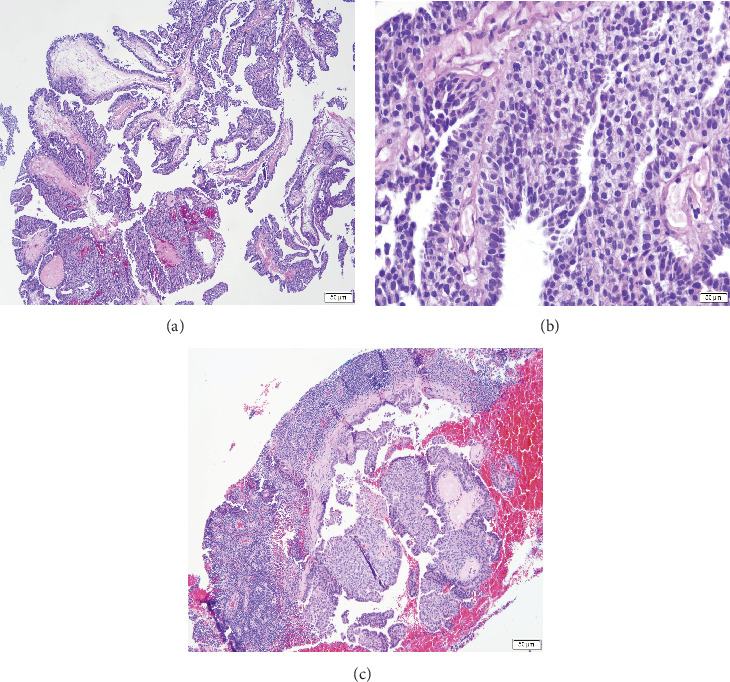
Histologic sections show (a) invasive carcinoma with papillary features and (b) increased mitotic figures. (c) A needle-core biopsy of the left axillary node shows metastatic carcinoma.

Concurrent needle core biopsies of his left breast and left axillary lymph node showed invasive carcinoma with papillary features (Figure 2a), histologic Grade 1 (Nottingham histologic score) but mitotically active (Figure 2b), estrogen receptor/progesterone receptor positive (100%/100%), and Her2 immunohistochemical stain negative (1+) with lymph node metastasis (Figure 2c), respectively.

CT scans of the abdomen, pelvis, and bone were done to complete staging, and the patient was clinically staged as a Stage III-B, T4 N1a M0.

**Figure 3:**
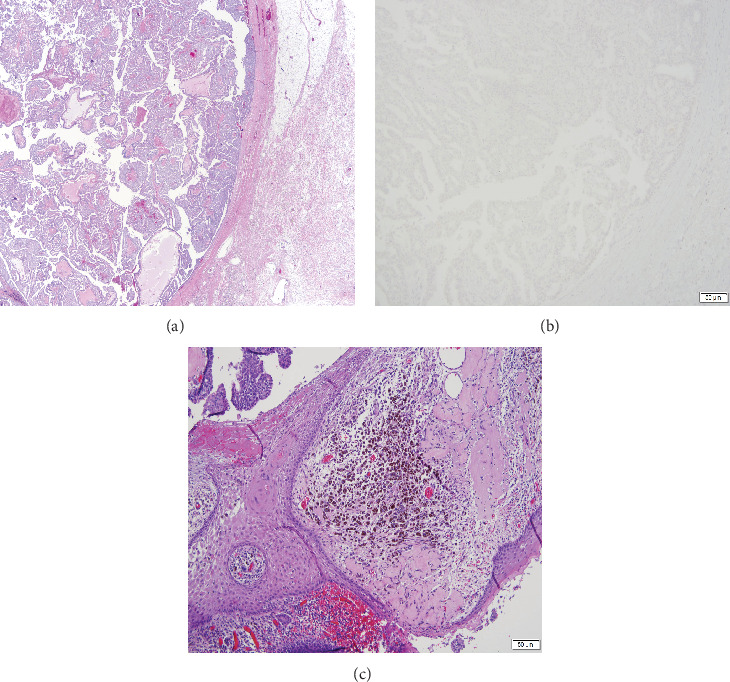
(a) Histologic sections show a well-circumscribed, fibrotic capsule surrounding tumor growing as delicate papillary fronds. (b) A p63 is negative, confirming the lack of myoepithelial cells within and surrounding the tumor. (c) Tumor (upper left corner) is associated with ulcerated skin.

He underwent neoadjuvant chemotherapy with adriamycin, cyclophosphamide, and paclitaxel followed by left radical mastectomy and scout-guided axillary dissection. The main specimen revealed EPC (Figure 3a), confirmed by lack of myoepithelial cells (p63 negative within and surrounding the tumor) (Figure 3b) and by the lack of neuroendocrine differentiation, which made the diagnosis of solid papillary carcinoma less likely. Margin status was as follows: present < 1 mm from the skin, 8 mm from the posterior margin, and more than 10 mm from all other margins. There was no definite response to presurgical therapy with an overall cancer cellularity of 95%. Additionally, there was associated skin ulceration (Figure 3c), with a final pathologic stage of ypT4bN1a.

Following surgical management, the patient completed a course of radiation therapy and initiated tamoxifen. Three years out, the patient is clinically doing well without evidence of disease recurrence.

## 3. Discussion

Approximately 1% of breast cancer diagnoses occur in male patients [1]. Papillary lesions, including EPC, may be relatively more common in male patients than other breast carcinomas, with 4% of papillary carcinomas and 3.5% of EPCs diagnosed in male patients [9, 11]. Breast cancer in men is often diagnosed at a later stage than in their female counterparts. Proposed explanations for this phenomenon include decreased awareness of breast cancer in men, no routine preventive screening, as well as a relative paucity of mammary tissue leading to rapid invasion of adjacent structures [12].

EPCs are pathologically staged as carcinoma in situ (pTis) in the absence of overt histologic invasion, which must be carefully differentiated from entrapped neoplastic cells within the fibrous capsule. The present case is unusual in that although no invasion was identified in the mastectomy specimen, the encapsulated tumor was associated with skin ulceration. Invasive tumor was noted in the patient's core needle specimen, rendering a differential diagnosis of a pure papillary carcinoma versus IDC with papillary features; ultimately, definitive classification was deferred to the resection specimen. Review of the biopsy specimen showed almost exclusively papillary lesional tissue without a significant amount of breast parenchyma to evaluate the presence of definitive stromal invasion, demonstrating the difficulty in diagnosing EPC on a limited biopsy sample [13].

While previous studies have suggested that sampling error could explain the presence of positive lymph nodes in pure EPC, the patient's lesion was extensively sampled (in its near entirety) with additional sections including grossly uninvolved breast tissue, without identification of an invasive component [2, 14]. To our knowledge, this is the only case in which EPC without associated invasion has been reported in association with skin ulceration in a male patient. This finding, as well as prior reports of positive lymph nodes identified in cases of pure EPC, may provide evidence for an invasive rather than in situ underlying pathophysiology of EPC [2, 14]. The lymph node metastases in our case recapitulated the architecture seen within the breast, with tumor cells in a papillary configuration entirely surrounded by a thick fibrous capsule.

Despite the overall excellent prognosis of EPC, several studies have shown that it is frequently associated with usual DCIS, IDC, or both [7, 15–17]. The clinical implications of these findings are uncertain. Larger series have shown a greater than 90% relative survival rate at 5 to 10 years even in patients who had concomitant invasive disease at the time of diagnosis [9].

The histologic type, both pure and associated with DCIS/invasion, as well as pathologic stage and sentinel lymph node status of EPCs reported in the male breast were reviewed in the English literature in PubMed (Table 1). Of the eighteen cases for which histologic information was available, ten were pure EPC (10/18, 55.6%), two were EPC with DCIS (2/18, 11.1%), and six were EPC with invasive, +/− DCIS (6/18, 33.3%). These results are consistent with studies with predominantly female patients with regard to the prevalence of an invasive component but show a relatively increased proportion of pure EPC in males when compared to prior observations in females [7, 15, 17].

Per our literature review, sentinel lymph nodes were either negative or not sampled in all cases of pure EPC with the exception of one case. Stolnicu et al. noted foci of subcapsular sinus tumor cells in the lymph nodes in association with groups of entrapped tumor cells within the prior biopsy site/needle track. They hypothesized that the tumor cells within the lymph nodes may have been a result of displacement of cells during the prior biopsy of the breast lesion due to the intrinsic friable nature of papillary lesions [21]. Eleven cases were either explicitly reported as stage pTis or implied as staged in situ; two cases were staged pT2 (one seemingly based upon the size of the intracystic component without invasion reported); and one case was staged as pT4 based upon an associated IDC. For 18 cases, including 14 cases of EPC as part of a larger 117 case series, staging information was not available for review [25].

Molecular profiling in multiple tumor types, including breast origin, is becoming routine practice with the rise of targeted therapies. Molecular analysis using next-generation sequencing (NGS) of six cases of EPC (three pure EPC and three EPC with associated invasive carcinoma) identified a similar molecular landscape, with four out of six total cases harboring PIK3CA hot spot mutation (all cases of EPC with invasive carcinoma and one of the cases of pure EPC). Notably, the single male patient in this series did not have a PIK3CA mutation [33]. These findings suggest that EPCs, with and without invasion, exhibit similarities at the molecular level, although larger studies would be required to better understand the molecular profile of these tumors.

Although guidelines for male patients with this rare tumor type are not clearly delineated, our patient subsequently underwent radiation and continues on tamoxifen therapy. Large studies in female patients with EPC have established a clear survival benefit in patients who received radiation therapy [34]. The vast majority of EPCs show estrogen receptor positivity by immunohistochemistry and therefore, many patients receive medications such as tamoxifen. However, the role of adjuvant endocrine therapy is not well defined in EPC [9, 34].

Even with advanced presentation, we document the apparent indolent biology of this tumor given the lack of locoregional or systemic recurrence 3 years from the primary diagnosis.

## 4. Conclusion

EPC is an uncommonly encountered tumor typically diagnosed in postmenopausal females, with rare case reports in the literature described in male patients. In general, it represents a less aggressive form of invasive carcinoma with a favorable prognosis and rare incidences of metastasis. Tumors with higher pathologic stage and lymph node/distant metastasis are usually associated with a concurrent overt invasive carcinoma. The present case highlights the metastatic potential of pure EPC. Furthermore, our case offers additional insight into the inherent biological indolence of EPC.

## Figures and Tables

**Table 1:** Histology, stage, and lymph node statuses of EPC in the male breast reported in the literature.

**Authors, year (reference)**	**Number of cases ( ** **N** ** )**	**Histologic type**	**Pathologic stage**	**Lymph node status**
Kinoshita et al., 2005 [18]	1	EPC	Tis	NS
Kinoshita et al., 2018 [19]	1	EPC with invasive	T2	−
Yılmaz et al., 2018[20]	1	EPC	T2^a^	−
Stolnicu et al., 2018[21]	1	EPC with DCIS	NR	+^b^
Mok et al., 2018 [22]	1	EPC	Tis	−
Singh et al., 2020 [23]	1	EPC with invasive	NR	−
Luo et al., 2020 [24]	3	1. EPC	1. Tis^c^	1. −
		2. EPC with invasive	2. NR	2. −
		3. EPC	3. Tis^c^	3. NS
Zhong et al., 2020[25]	14	EPC^d^	NR^e^	NS/−^f^
Chen et al., 2022[26]	1	EPC	Tis^c^	NS
Huang et al., 2022[10]	1	EPC with invasive	T4	+
Li et al., 2022 [13]	1	EPC	Tis	−
Avau et al., 2022[27]	1	EPC with invasive^g^	N/A	+
Hong et al., 2023[28]	1	EPC, DCIS, and invasive	NR	−
Yu et al., 2023[29]	1	EPC	Tis^c^	NS
Wang et al., 2023[30]	1	EPC with DCIS	Tis^c^	NS
Sugino et al., 2023[31]	1	EPC	Tis	NS
Gogoi et al., 2023[32]	1^h^	EPC	Tis^c^	−

Abbreviations: −: negative; +: positive; NS: not sampled; NR: not reported.

^a^This case was reported only as EPC without observed invasion; however, the reported stage was T2, possibly reflecting recent changes within the WHO guidelines of staging for these tumors based.

^b^No invasive component was identified in this case; however, foci of tumor cells within the subcapsular sinus were noted in two sentinel lymph nodes.

^c^This report did not explicitly report the stage as Tis; however, the invasion was not reported and the stage of Tis was assumed based on pure EPC/EPC with only DCIS based on the WHO staging guidelines.

^d^117 cases of papillary neoplasms, including 14 cases of EPC that included cases with microinvasive carcinoma.

^e^Staging information not explicitly provided for each case of EPC.

^f^Out of all neoplastic papillary lesions evaluated, 63% had axillary lymph node sampling and only two cases had metastases, neither of which were cases of EPC. However, it is unclear whether all cases of EPC had axillary sampling.

^g^Bilateral EPCs, with bilateral DCIS and unilateral invasive carcinoma.

^h^Six papillary neoplasms were reviewed of any sex, with one case of EPC which was identified in the only male patient in the case series.

## Data Availability

The figures and table supporting the findings of this case study are available within the article.
